# Redetermination of terbium scandate, revealing a defect-type perovskite derivative

**DOI:** 10.1107/S1600536808033394

**Published:** 2008-10-31

**Authors:** Boža Veličkov, Volker Kahlenberg, Rainer Bertram, Reinhard Uecker

**Affiliations:** a Leibniz-Institute for Crystal Growth, Max-Born-Strasse 2, 12489 Berlin, Germany; b University of Innsbruck, Institute of Mineralogy and Petrography, Innrain 52, 6020 Innsbruck, Austria

## Abstract

The crystal structure of terbium(III) scandate(III), with ideal formula TbScO_3_, has been reported previously on the basis of powder diffraction data [Liferovich & Mitchell (2004 ▶). *J. Solid State Chem.* **177**, 2188–2197]. The current data were obtained from single crystals grown by the Czochralski method and show an improvement in the precision of the geometric parameters. Moreover, inductively coupled plasma optical emission spectrometry studies resulted in a nonstoichiometric composition of the title compound. Site-occupancy refinements based on diffraction data support the idea of a Tb deficiency on the *A* site (inducing O defects on the O2 position). The crystallochemical formula of the investigated sample thus may be written as ^*A*^(□_0.04_Tb_0.96_)^*B*^ScO_2.94_. In the title compound, Tb occupies the eightfold-coordinated sites (site symmetry *m*) and Sc the centres of corner-sharing [ScO_6_] octa­hedra (site symmetry 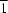). The mean bond lengths and site distortions fit well into the data of the remaining lanthanoid scandates in the series from DyScO_3_ to NdScO_3_. A linear structural evolution with the size of the lanthanoid from DyScO_3_ to NdScO_3_ can be predicted.

## Related literature

Rietvelt refinements on powders of LnScO_3_ with Ln = La^3+^–Ho^3+^ were reported by Liferovich & Mitchell (2004 ▶). The crystal structures of the Dy, Gd, Sm and Nd members, refined from single-crystal diffraction data, have been recently provided by Veličkov *et al.* (2007 ▶). Geometrical parameters have been calculated by means of atomic coordinates following the concept of Zhao *et al.* (1993 ▶). A more detailed description of the growth procedure of the Ln scandates is given by Uecker *et al.* (2006 ▶). For the applications of Ln scandates, see: Choi *et al.* (2004 ▶); Haeni *et al.* (2004 ▶).

## Experimental

### 

#### Crystal data

Tb_0.96_ScO_2.94_
*M* *_r_* = 244.56
Orthorhombic, 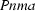

*a* = 5.7233 (8) Å
*b* = 7.9147 (12) Å
*c* = 5.4543 (7) Å
*V* = 247.07 (6) Å^3^
*Z* = 4
Mo *K*α radiation
μ = 29.58 mm^−1^
*T* = 298 (2) K
0.14 × 0.12 × 0.02 mm

#### Data collection

Stoe IPDS-II diffractometer
Absorption correction: analytical (Alcock, 1970 ▶) *T*_min_ = 0.088, *T*_max_ = 0.278
2143 measured reflections
353 independent reflections
328 reflections with *I* > 2σ(*I*)
*R*_int_ = 0.065

#### Refinement

*R*[*F* ^2^ > 2σ(*F* ^2^)] = 0.024
*wR*(*F* ^2^) = 0.047
*S* = 1.20
353 reflections
31 parameters
1 restraint
Δρ_max_ = 2.15 e Å^−3^
Δρ_min_ = −1.12 e Å^−3^

### 

Data collection: *X-AREA* (Stoe & Cie, 2006 ▶); cell refinement: *X-AREA*; data reduction: *X-RED32* (Stoe & Cie, 2006 ▶); program(s) used to solve structure: *SHELXS97* (Sheldrick, 2008 ▶); program(s) used to refine structure: *SHELXS97* (Sheldrick, 2008 ▶); molecular graphics: *ATOMS* (Dowty, 2004 ▶); software used to prepare material for publication: *SHELXL97*.

## Supplementary Material

Crystal structure: contains datablocks I, global. DOI: 10.1107/S1600536808033394/wm2190sup1.cif

Structure factors: contains datablocks I. DOI: 10.1107/S1600536808033394/wm2190Isup2.hkl

Additional supplementary materials:  crystallographic information; 3D view; checkCIF report

## Figures and Tables

**Table 1:** Selected bond lengths (Å)

Tb1—O1^i^	2.241 (5)
Tb1—O2^ii^	2.277 (4)
Tb1—O1^iii^	2.334 (5)
Tb1—O2^iv^	2.586 (4)
Tb1—O2^v^	2.837 (4)
Sc2—O2^ii^	2.088 (3)
Sc2—O2^vi^	2.095 (4)
Sc2—O1^vii^	2.1141 (19)

Symmetry codes: (i) 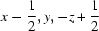; (ii) 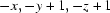; (iii) 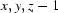; (iv) 
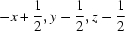
; (v) 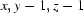; (vi) 
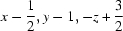
; (vii) 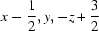.

**Fig. 1.**
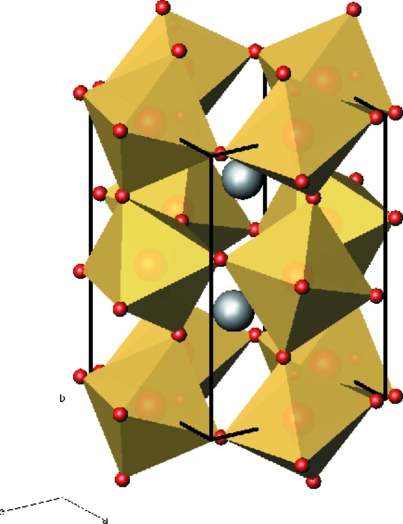
The orthorhombic perovskite structure of TbScO3 characterized by a tilted corner sharing ScO6 framework and the 8-fold coordinated Tb sites. The ScO6 octahedra are brownish and translucent, the Tb atoms are grey and the O atoms are red.

**Fig. 2.**
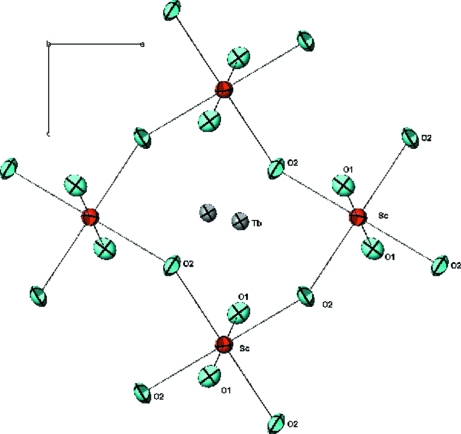
Projection of the TbScO3 structure along [010], showing the Tb atoms and the Sc coordination with displacement ellipsoids at the 80% probability level.

## supplementary crystallographic information

### Comment

The lanthanoid scandates, LnScO_3_, with Ln = La^3+^ to Ho^3+^ are known to adopt an orthorhombic derivative of the perovskite structure. Their lattice dimensions are suitable to use them as substrates for the epitaxial growth of strain engineered BaTiO_3_ and SrTiO_3_ films (Choi *et al.*, 2004; Haeni *et al.*, 2004).

Liferovich & Mitchell (2004) studied the crystal structure of lanthanoid scandates, including TbScO_3_, by Rietveld analysis from powder diffraction data. Crystallographic data of DyScO_3_, GdScO_3_, SmScO_3_ and NdScO_3_ obtained from single crystals were recently reported by Veličkov *et al.* (2007). However, in the literature there are disagreements concerning some structural characteristics and their dependence on the Ln-substitution: Veličkov *et al.* (2007) assumed linear trends, whereas Liferovich & Mitchell (2004) observed no obvious continious evolution. Especially the TbScO_3_ and EuScO_3_ compounds seemed to exhibit an anomalous behaviour in the latter study. The present paper provides first results on TbScO_3_, redetermined from single-crystal data. Investigations on EuScO_3_ are in preparation.

The orthorhombic distorted perovskite structure of TbScO_3_ (Fig.1) is confirmed from our refinements. Whereas the lattice parameters for TbScO_3_ compare well with the data of Liferovich & Mitchell (2004), the atomic coordinates show deviations of up to 0.008 in the fractional atomic coordinates, resulting in slightly different geometrical parameters. The A-site is occupied by Tb and has an average bond length in an eightfold coordination of ^[8]^<A—O> = 2.499 Å with a polyhedral bond length distortion of ^A^Δ_8_ = 8.78x10^-3 ^(Δ_n_=1/nΣ{(r_i_-r)/r}^2^). The B-site shows bond lengths typical for octahedrally coordinated scandium (<B—O> = 2.101 Å) and is rather distorted with ^B^Δ_6_ = 0.025x10^-3^ and a bond angle variance of δ = 3.23°. The tilting of the corner sharing octahedra calculated after Zhao *et al.* (1993) are θ = 20.64° in [110] and Ø = 12.97° in [001] directions. From our data we can establish linear trends for the crystallochemical parameters from DyScO_3_ to NdScO_3_ in dependence on the Ln-substitution. Consequently, an anomalous behaviour of TbScO_3_ in Ln-scandate series could not be confirmed.

### Experimental

TbScO_3_ was grown as a bulk crystal (Ø = 20 mm) from a melt by conventional Czochralski technique with an automatic diameter control. The starting materials Tb_4_O_7 _and Sc_2_O_3_ (Alfa Aesar) with 99.99% purity were dried, mixed in a stoichiometric ratio, sintered and pressed to pellets easing the melting procedure. An iridium crucible (40 *x* 40 mm) was used as melt container combined with an iridium afterheater both RF-heated with a 25 kW mf generator. The crystal was withdrawn with a pulling rate of 1 mm/h under flowing nitrogen atmosphere. The grown crystal was colourless, so that a valence state of Tb^3+^ can be assumed. A part of the single-crystal material was crushed and irregular fragments were screened using a polarizing light microscope to find a sample of good optical quality for diffraction experiments.

### Refinement

The ICP OES (inductively coupled plasma optical emission spectrometry) investigation of this sample resulted in a compostion of Tb_2_O_3_ = 48.79 mol% and Sc_2_O_3 _= 51.21 mol%, indicating a non-stoichiometric chemical composition. Site occupancy refinements based on diffraction data support the idea of the Tb-deficiency on the A-site coupled with O-defects on the O2-position. The calculated chemical compositions provided by structure refinement agree very well with the data of the ICP OES study. The crystallochemical formula of the investigated sample may thus be written as ^A^(□_0.04_Tb_0.96_)^B^ScO_2.94._

The highest peak and deepest hole are located 0.59 and 1.42 Å from Tb1. Site occupation refinements indicated deviations from full occupancy on the Tb1 and the O2 sites. For the final refinement cycle a constraint ensuring charge neutrality was included. In contrast to the previous powder refinement, performed with the setting *Pbnm* of space group no. 62, the standard setting in *Pnma* was used for the present redetermination.

### Crystal data

**Table:**

Tb_0.96_ScO_2.94_	*F*(000) = 427
*M**_r_* = 244.56	*D*_x_ = 6.55 Mg m^−^^3^
Orthorhombic, *P**n**m**a*	Mo *K*α radiation, λ = 0.71073 Å
Hall symbol: -P 2ac 2n	Cell parameters from 1947 reflections
*a* = 5.7233 (8) Å	θ = 2.6–29.1°
*b* = 7.9147 (12) Å	µ = 29.58 mm^−^^1^
*c* = 5.4543 (7) Å	*T* = 298 K
*V* = 247.07 (6) Å^3^	Plate, colourless
*Z* = 4	0.14 × 0.12 × 0.02 mm

### Data collection

**Table:**

Stoe IPDS-II diffractometer	353 independent reflections
Radiation source: fine-focus sealed tube	328 reflections with *I* > 2σ(*I*)
graphite	*R*_int_ = 0.065
Detector resolution: 6.67 pixels mm^-1^	θ_max_ = 29.1°, θ_min_ = 4.5°
ω scans	*h* = −7→7
Absorption correction: analytical (Alcock, 1970)	*k* = −9→10
*T*_min_ = 0.088, *T*_max_ = 0.278	*l* = −7→7
2143 measured reflections	

### Refinement

**Table:**

Refinement on *F*^2^	Primary atom site location: structure-invariant direct methods
Least-squares matrix: full	Secondary atom site location: difference Fourier map
*R*[*F*^2^ > 2σ(*F*^2^)] = 0.024	*w* = 1/[σ^2^(*F*_o_^2^) + (0.0165*P*)^2^ + 1.3905*P*] where *P* = (*F*_o_^2^ + 2*F*_c_^2^)/3
*wR*(*F*^2^) = 0.047	(Δ/σ)_max_ = 0.015
*S* = 1.20	Δρ_max_ = 2.15 e Å^−^^3^
353 reflections	Δρ_min_ = −1.11 e Å^−^^3^
31 parameters	Extinction correction: SHELXS97 (Sheldrick, 2008), Fc^*^=kFc[1+0.001xFc^2^λ^3^/sin(2θ)]^-1/4^
1 restraint	Extinction coefficient: 0.158 (6)

### Special details

**Table:**

Geometry. All e.s.d.'s (except the e.s.d. in the dihedral angle between two l.s. planes) are estimated using the full covariance matrix. The cell e.s.d.'s are taken into account individually in the estimation of e.s.d.'s in distances, angles and torsion angles; correlations between e.s.d.'s in cell parameters are only used when they are defined by crystal symmetry. An approximate (isotropic) treatment of cell e.s.d.'s is used for estimating e.s.d.'s involving l.s. planes.
Refinement. Refinement of *F*^2^ against ALL reflections. The weighted *R*-factor *wR* and goodness of fit *S* are based on *F*^2^, conventional *R*-factors *R* are based on *F*, with *F* set to zero for negative *F*^2^. The threshold expression of *F*^2^ > σ(*F*^2^) is used only for calculating *R*-factors(gt) *etc*. and is not relevant to the choice of reflections for refinement. *R*-factors based on *F*^2^ are statistically about twice as large as those based on *F*, and *R*- factors based on ALL data will be even larger.

### Fractional atomic coordinates and isotropic or equivalent isotropic displacement parameters (Å^2^)

**Table:**

	*x*	*y*	*z*	*U*_iso_*/*U*_eq_	Occ. (<1)
Tb1	0.06029 (6)	0.25	0.01672 (6)	0.0087 (2)	0.9591 (13)
Sc2	0	0	0.5	0.0082 (3)	
O1	0.4455 (10)	0.25	0.8761 (9)	0.0114 (10)	
O2	0.1946 (7)	0.9357 (5)	0.8100 (6)	0.0108 (8)	0.9693 (10)

### Atomic displacement parameters (Å^2^)

**Table:**

	*U*^11^	*U*^22^	*U*^33^	*U*^12^	*U*^13^	*U*^23^
Tb1	0.0074 (3)	0.0106 (3)	0.0080 (2)	0	0.00053 (12)	0
Sc2	0.0085 (6)	0.0085 (7)	0.0075 (5)	−0.0003 (7)	−0.0002 (4)	0.0002 (4)
O1	0.013 (3)	0.010 (2)	0.012 (2)	0	0.0018 (19)	0
O2	0.0078 (19)	0.014 (2)	0.0111 (15)	−0.0024 (14)	−0.0037 (13)	0.0025 (13)

### Geometric parameters (Å, °)

**Table:**

Tb1—O1^i^	2.241 (5)	Sc2—O2^ii^	2.088 (3)
Tb1—O2^ii^	2.277 (4)	Sc2—O2^xii^	2.088 (3)
Tb1—O2^iii^	2.277 (4)	Sc2—O2^xiii^	2.095 (4)
Tb1—O1^iv^	2.334 (5)	Sc2—O2^vi^	2.095 (4)
Tb1—O2^v^	2.586 (4)	Sc2—O1^xiv^	2.1141 (19)
Tb1—O2^vi^	2.586 (4)	Sc2—O1^x^	2.1141 (18)
Tb1—O2^vii^	2.837 (4)	Sc2—Tb1^xv^	3.2026 (4)
Tb1—O2^viii^	2.837 (4)	Sc2—Tb1^i^	3.2026 (4)
Tb1—Sc2^ix^	3.2026 (4)	Sc2—Tb1^xvi^	3.3140 (4)
Tb1—Sc2^x^	3.2026 (4)	Sc2—Tb1^xvii^	3.4608 (4)
Tb1—Sc2^xi^	3.3140 (4)	Sc2—Tb1^xviii^	3.4608 (4)
Tb1—Sc2	3.3140 (4)		
			
O1^i^—Tb1—O2^ii^	102.07 (14)	O2^xii^—Sc2—O2^xiii^	89.16 (7)
O1^i^—Tb1—O2^iii^	102.07 (14)	O2^ii^—Sc2—O2^vi^	89.16 (7)
O2^ii^—Tb1—O2^iii^	80.4 (2)	O2^xii^—Sc2—O2^vi^	90.84 (7)
O1^i^—Tb1—O1^iv^	87.86 (12)	O2^xiii^—Sc2—O2^vi^	180
O2^ii^—Tb1—O1^iv^	137.88 (11)	O2^ii^—Sc2—O1^xiv^	87.26 (17)
O2^iii^—Tb1—O1^iv^	137.88 (11)	O2^xii^—Sc2—O1^xiv^	92.74 (17)
O1^i^—Tb1—O2^v^	138.63 (11)	O2^xiii^—Sc2—O1^xiv^	86.91 (18)
O2^ii^—Tb1—O2^v^	117.25 (8)	O2^vi^—Sc2—O1^xiv^	93.09 (18)
O2^iii^—Tb1—O2^v^	73.97 (9)	O2^ii^—Sc2—O1^x^	92.74 (17)
O1^iv^—Tb1—O2^v^	72.00 (13)	O2^xii^—Sc2—O1^x^	87.26 (17)
O1^i^—Tb1—O2^vi^	138.63 (11)	O2^xiii^—Sc2—O1^x^	93.09 (18)
O2^ii^—Tb1—O2^vi^	73.97 (9)	O2^vi^—Sc2—O1^x^	86.91 (18)
O2^iii^—Tb1—O2^vi^	117.25 (8)	O1^xiv^—Sc2—O1^x^	180
O1^iv^—Tb1—O2^vi^	72.00 (13)	Sc2^xix^—O1—Sc2^xv^	138.8 (3)
O2^v^—Tb1—O2^vi^	69.28 (17)	Sc2^xix^—O1—Tb1^xx^	105.22 (14)
O1^i^—Tb1—O2^vii^	72.51 (9)	Sc2^xv^—O1—Tb1^xx^	105.22 (14)
O2^ii^—Tb1—O2^vii^	76.86 (13)	Sc2^xix^—O1—Tb1^xviii^	91.96 (15)
O2^iii^—Tb1—O2^vii^	154.79 (10)	Sc2^xv^—O1—Tb1^xviii^	91.96 (15)
O1^iv^—Tb1—O2^vii^	67.26 (9)	Tb1^xx^—O1—Tb1^xviii^	126.2 (2)
O2^v^—Tb1—O2^vii^	126.67 (6)	Sc2^xxi^—O2—Sc2^xxii^	141.9 (2)
O2^vi^—Tb1—O2^vii^	66.45 (5)	Sc2^xxi^—O2—Tb1^ii^	98.72 (15)
O1^i^—Tb1—O2^viii^	72.51 (9)	Sc2^xxii^—O2—Tb1^ii^	119.09 (16)
O2^ii^—Tb1—O2^viii^	154.79 (10)	Sc2^xxi^—O2—Tb1^xxii^	85.81 (12)
O2^iii^—Tb1—O2^viii^	76.86 (13)	Sc2^xxii^—O2—Tb1^xxii^	89.52 (13)
O1^iv^—Tb1—O2^viii^	67.26 (9)	Tb1^ii^—O2—Tb1^xxii^	103.74 (15)
O2^v^—Tb1—O2^viii^	66.45 (5)	Sc2^xxi^—O2—Tb1^xxiii^	87.91 (13)
O2^vi^—Tb1—O2^viii^	126.67 (6)	Sc2^xxii^—O2—Tb1^xxiii^	79.43 (12)
O2^vii^—Tb1—O2^viii^	122.50 (15)	Tb1^ii^—O2—Tb1^xxiii^	103.14 (13)
O2^ii^—Sc2—O2^xii^	180	Tb1^xxii^—O2—Tb1^xxiii^	153.02 (16)
O2^ii^—Sc2—O2^xiii^	90.84 (7)		

Symmetry codes: (i) *x*−1/2, *y*, −*z*+1/2; (ii) −*x*, −*y*+1, −*z*+1; (iii) −*x*, *y*−1/2, −*z*+1; (iv) *x*, *y*, *z*−1; (v) −*x*+1/2, *y*−1/2, *z*−1/2; (vi) −*x*+1/2, −*y*+1, *z*−1/2; (vii) *x*, *y*−1, *z*−1; (viii) *x*, −*y*+3/2, *z*−1; (ix) *x*+1/2, −*y*+1/2, −*z*+1/2; (x) −*x*+1/2, −*y*, *z*−1/2; (xi) −*x*, *y*+1/2, −*z*+1; (xii) *x*, *y*−1, *z*; (xiii) *x*−1/2, *y*−1, −*z*+3/2; (xiv) *x*−1/2, *y*, −*z*+3/2; (xv) −*x*+1/2, −*y*, *z*+1/2; (xvi) −*x*, −*y*, −*z*+1; (xvii) −*x*, −*y*, −*z*; (xviii) *x*, *y*, *z*+1; (xix) *x*+1/2, −*y*+1/2, −*z*+3/2; (xx) *x*+1/2, *y*, −*z*+1/2; (xxi) *x*, *y*+1, *z*; (xxii) −*x*+1/2, −*y*+1, *z*+1/2; (xxiii) *x*, *y*+1, *z*+1.
